# Rapid Insulinotropic Action of Low Doses of Bisphenol-A on Mouse and Human Islets of Langerhans: Role of Estrogen Receptor β

**DOI:** 10.1371/journal.pone.0031109

**Published:** 2012-02-08

**Authors:** Sergi Soriano, Paloma Alonso-Magdalena, Marta García-Arévalo, Anna Novials, Sarheed J. Muhammed, Albert Salehi, Jan-Ake Gustafsson, Ivan Quesada, Angel Nadal

**Affiliations:** 1 Instituto Bioingeniería and CIBER de Diabetes y Enfermedades Metabólicas Asociadas (CIBERDEM), Universidad Miguel Hernández de Elche, Elche, Alicante, Spain; 2 Institut D'Investigacions Biomèdiques August Pi i Sunyer (IDIBAPS) and CIBERDEM, Barcelona, Spain; 3 Department of Clinical Sciences, Lund University, Malmö, Sweden; 4 Department of Cell Biology and Biochemistry, Center for Nuclear Receptors and Cell Signaling, University of Houston, Houston, Texas, United States of America; University of Bremen, Germany

## Abstract

Bisphenol-A (BPA) is a widespread endocrine-disrupting chemical (EDC) used as the base compound in the manufacture of polycarbonate plastics. It alters pancreatic β-cell function and can be considered a risk factor for type 2 diabetes in rodents. Here we used ERβ−/− mice to study whether ERβ is involved in the rapid regulation of K_ATP_ channel activity, calcium signals and insulin release elicited by environmentally relevant doses of BPA (1 nM). We also investigated these effects of BPA in β-cells and whole islets of Langerhans from humans. 1 nM BPA rapidly decreased K_ATP_ channel activity, increased glucose-induced [Ca^2+^]_i_ signals and insulin release in β-cells from WT mice but not in cells from ERβ−/− mice. The rapid reduction in the K_ATP_ channel activity and the insulinotropic effect was seen in human cells and islets. BPA actions were stronger in human islets compared to mouse islets when the same BPA concentration was used. Our findings suggest that BPA behaves as a strong estrogen via nuclear ERβ and indicate that results obtained with BPA in mouse β-cells may be extrapolated to humans. This supports that BPA should be considered as a risk factor for metabolic disorders in humans.

## Introduction

Bisphenol-A (BPA) is a widespread endocrine disruptor that produces insulin resistance and alterations in pancreatic β-cell function [1]. It has been suggested that together with other endocrine disrupting chemicals (EDCs), BPA constitutes a risk factor for type 2 diabetes and other metabolic disorders [2]–[4]. Recent work shows the association between increasing urinary BPA levels and Diabetes Mellitus. A 12.8% of diabetic patients show high BPA levels in urine samples (4.20 ng/ml or 18 nM) [5]. Moreover an association between urinary levels of BPA, obesity and insulin resistance in middle-aged and elderly chinese adults has been recently described [6]. The range of BPA levels found in humans is from 0.7 to 20 nM [7], [8]. To support the evidence that BPA may be a risk for the development of diabetes it is critical to study its effect on human tissues involved in glucose and lipid metabolism, including the endocrine pancreas which is key in glucose homeostasis.

The islet of Langerhans is the physiological unit of the endocrine pancreas; it is a group of 1500–3000 cells of five different types [9]–[11], and the most abundant are β-cells. The main function of β-cells is the biosynthesis and release of insulin in response to neurotransmitters, hormones and nutrients, the most important being glucose. The secretory response of β-cells depends on their electrical activity. This consists of oscillations of the membrane potential that range from electrically silent periods to depolarized plateaus on which Ca^2+^-action potential originate [12]. Classically, stimulus-secretion coupling involves the closure of K_ATP_ channels after an increase in the ATP/ADP ratio because of the glucose metabolism [13]. K_ATP_ channels are responsible for the resting membrane potential of β-cells and its closure elicits a depolarization that opens voltage dependent calcium channels and induces Ca^2+^ influx [14]. As a consequence of the oscillatory membrane potential, a [Ca^2+^]_i_ oscillatory pattern originates [15]–[19], which triggers a pulsatile insulin secretion [17]–[19]. In addition to the K_ATP_ dependent process of insulin secretion there is a K_ATP_ independent process which involves cAMP dependent phosphorylation [20], [21].

Beta cells express estrogen receptor α (ERα), estrogen receptor β (ERβ) and the G-protein coupled receptor (GPR30), also named GPER1 [22], [23]. The use of genetically modified mice has revealed the role of these estrogen receptors [23]. ERα is involved in the regulation of pancreatic insulin biosynthesis in response to both E2 and BPA. Remarkably, nanomolar concentrations of either BPA or E2 act via extranuclear ERα to activate ERK1/2 and regulate insulin content [24]. This action involves the activation of the transcription factor NeuroD1 [25]. In addition, activation of extranuclear ERβ by physiological concentrations of E2 rapidly regulates K_ATP_ channel activity, increases glucose stimulated [Ca^2+^]_i_ signals and insulin release. It is important to clarify that action of E2 on K_ATP_ channel activity was mimicked by specific agonist of ERβ 2,3-bis (4-hydroxyphenyl)-propionitrile (DPN) but not by the ERα specific agonist propylpyrazole-triol (PPT). Moreover K_ATP_ channel activity was not modified in ERα−/− [26]. These results indicated that ERβ plays an important role in rapid regulation of insulin secretion in pancreatic β-cells. Bisphenol-A imitated rapid estradiol regulation of K_ATP_ channel and calcium signaling [1], however, whether ERβ acting out of the nucleus is able to mediate BPA actions is still unknown.

In the present work we use β-cells and islets of Langerhans from wild type (WT) and ERβ−/− mice to show that ERβ is involved in the BPA-mediated rapid regulation of K_ATP_ channel activity, potentiation of glucose induced-[Ca^2+^]_i_ signals and insulin release. Moreover, we have used human islets of Langerhans to demonstrate that 1 nM BPA blocked K_ATP_ channels and produced a potent enhancement of insulin secretion in response to glucose.

## Materials and Methods

### Animals

Adult C57 female mice were used. All animals were kept under standard housing conditions. The ethical committee of Miguel Hernandez University “Comisión de Ética en la Investigación Experimental” reviewed and approved the method used. ERβ−/−mice were generated as described prior [27] and supplied by Jan-Ake Gustafsson's laboratory. All genetically modified animals and wild types were from the same supplier and the same colony. Islets of Langerhans from ERβ−/− mice were treated as described below for islets from C57 mice. Animals were kept in new polycarbonate cages and polycarbonate water bottles were avoided to minimize contamination of mice with free BPA.

### Islet of Langerhans isolation from mice

Adult mice were killed by cervical dislocation and islets were isolated with a collagenase technique previously described [26] and used according to the kind of experiment to be performed.

### Human islets isolation for electrophysiological experiments

Human pancreases were obtained from 3 cadaveric organ donors, after written consent of their families and approval of “Comité Ético de Investigación Clínica”, and “Comité de Investigación”, Hospital Clínic of Barcelona (ID: 2009/5157). Islets were isolated by collagenase digestion of the pancreas (SERVA Electrophoresis, Heidelberg, Germany) and separated from exocrine tissue by Biocoll density gradient (Biochrom, Berlin,Germany), as previously described [28]. Islets were transferred to RPMI-1640 medium (Gibco-BRL, Pisley, U.K.) containing 11.1 mmol/l glucose and supplemented with 10% FBS, 2 mmol/l L-glutamine, 100 units/ml penicillin, and 100 µg/ml streptomycin and cultured overnight at 37°C with 5% CO2. Islets were then picked and disaggregated to obtain beta cells for electrophysiological studies as described below.

### Obtaining β-cells from mouse and human islets

Once mouse or human islets were obtained as described above, they were dispersed into single cells and cultured as previously described [14]. The protocol of islet disaggregation into sinlge cells was the same for both mouse and human islets. Once isolated, islets were disaggregated into single cells in a low calcium medium. Cells were then centrifuged, resuspended in culture medium RPMI 1640 without phenol red containing 11 mM glucose supplemented with 10% charcoal dextran-treated fetal bovine serum (HyClone Laboratories, Logan, UT), 2 mM L-glutamine, 200 U/mL penicillin and 0.2 mg/mL streptomycin and plated on glass coverslips. Cells were kept at 37°C, in a humidified atmosphere of 95% O_2_ and 5% CO_2_, and used within 1–2 days of culture.

### Insulin secretion measurements in mouse islets

Freshly isolated islets of Langerhans from mice were left to recover in the isolation medium for 2 h in the incubator. After recovery, groups of 5 were transferred to 400 µl of a buffer solution containing 140 mM NaCl, 4.5 mM KCl, 2.5 mM CaCl2, 1 mM MgCl2, 20 mM HEPES and the glucose concentration corresponding to each experimental condition with final pH at 7.4. Afterwards, 100 µl of the corresponding buffer solution with 5% Bovine Serum Albumin (BSA) was added, incubated at room temperature for 3 min and let to cool down for 15 min on ice. Then, the medium was collected and insulin was measured in duplicate samples by radioimmunoassay using a Coat-a-Count kit (Siemens, Los Angeles, CA, USA).

### Recording intracellular calcium concentration ([Ca^2+^]_i_)

Freshly isolated islets of Langerhans from mice were loaded with 5 µM Fura-2 AM for at least 1 h at room temperature. Calcium recordings in islets were obtained by imaging intracellular calcium under an inverted epifluorescence microscope (Zeiss, Axiovert 200). Images were acquired every 2 s with an extended Hamamatsu Digital Camera C4742-95 (Hamamatsu Photonics, Barcelona, Spain) using a dual filter wheel (Sutter Instrument CO, CA, USA) equipped with 340 nm and 380 nm, 10 nm bandpass filters (Omega optics, Madrid, Spain). Data were acquired using Aquacosmos software from Hamamatsu (Hamamatsu Photonics, Barcelona, Spain). Results were plotted and analyzed using commercially available software (Sigmaplot, Jandel Scientific). Data were represented as a frequency of calcium oscillations (min^−1^) comparing BPA responses with their respective control.

### Patch Clamp recordings

K_ATP_ channel activity was recorded using standard patch clamp recording procedures from isolated β-cells [29]. 80–90% of the single cells were identified as β-cells by their large size (capacitance: 8–12 pF) and by their electrophysiological properties, such as presence of K_ATP_ channel activity in absence of glucose and their response to glucose by the appearance of action currents in cell-attached configuration. Currents were recorded using an Axopatch 200B patch-clamp amplifier (Axon Instruments Co. CA, USA). Patch pipettes were pulled from borosilicate capillaries (Sutter Instruments Co. CA, USA) using a flaming/brown micropipette puller P-97 (Sutter Instruments Co. CA, USA) with resistance between 3–5 MΩ when filled with the pipette solutions as specified below. Bath solution contained (in mM): 5 KCl, 135 NaCl, 2.5 CaCl_2_, 10 Hepes and 1.1 MgCl_2_ (pH 7.4) and supplemented with glucose as indicated. The pipette solution contained (in mM): 140 KCl, 1 MgCl_2_, 10 Hepes and 1 EGTA (pH 7.2). The pipette potential was held at 0 mV throughout recording. K_ATP_ channel activity was quantified by digitising 60 s sections of the current record, filtered at 1 kHz, sampled at 10 kHz by a Digidata 1322A (Axon Instruments Co. CA, USA), and calculating the mean open probability of the channel (*NP_ o_*) during the sweep. Channel activity was defined as the product of *N*, the number of functional channels, and *P_o_*, the open-state probability. *P_o_* was determined by dividing the total time channels spent in the open state by the total sample time. Data were represented as a percentage of activity with respect to resting conditions (0 mM Glucose). Experiments were carried out at room temperature (20–24°C).

### Human pancreatic islets and measurement of human islet insulin secretion

Isolated human pancreatic islets from non-diabetic males (age 58+6 years; Hb1Ac<6.1 and MBI: 27+2) were provided by the Nordic network for clinical islet transplantation (Professor Olle Korsgren, Uppsala University, Sweden). All procedures were approved by the ethical committees at Uppsala and Lund Universities. Prior to the experiments the islets had been cultured at 37°C (5% CO_2_) for 2 days in CMRL 1066 (ICN Biomedicals, Costa Mesa, CA) supplemented with 10 mmol/l HEPES, 2 mmol/l L-glutamine, 50 µg/ml gentamicin, 0.25 µg/ml Fungizone (Gibco, BRL, Gaithersburg, MD), 20 µg/ml ciprofloxacin (Bayer Healthcare, Leverkusen, Germany) and 10 mmol/l nicotinamide. The islets had 70–90% purity when they arrived; the islets were then hand-picked under stereomicroscope prior to use. All islet preparations were treated in exactly the same way to avoid results being due to handling differences. All procedures were approved by the ethical committees at Uppsala and Lund Universities.

At the experiment day, the islets were preincubated for 30 min at 37°C in Krebs-Ringer bicarbonate buffer, pH 7.4, supplemented with 10 mmol/l HEPES, 0.1% bovine serum albumin and 1.0 mmol/l glucose. After preincubation the buffer was changed and the islets were incubated at 8.3 mmol/l glucose ± different test agents for 60 min at 37°C. Each incubation tube contained 12 islets in 1.0 ml of KRB solution and was gassed with 95% O_2_ 5% CO_2_ to obtain constant pH and oxygenation. All incubations were performed in an incubation box at 30 cycles/min. immediately after incubation an aliquot of the medium was removed and frozen for subsequent assay of insulin. The secreted insulin was measured using a radioimmunoassay kit (Millipore).

## Results

### BPA action on K_ATP_ channel activity is abolished in β-cells from ERβ−/− mice

**Figure 1:**
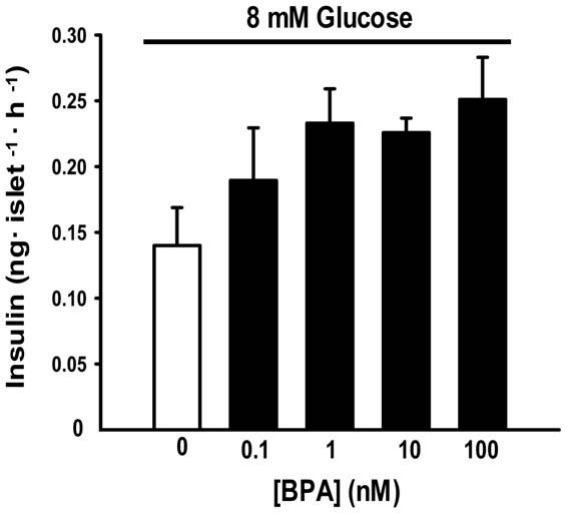
BPA increases insulin release in a dose dependent manner in mouse islet of langerhans. The released insulin was measured after one hour exposure of islets to increasing concentration of BPA, in the presence of 8 mM glucose. All points contained same amount of vehicle.

Bisphenol-A application in presence of 8 mM glucose during 1 hour increased insulin release from mouse islet of Langerhans in a dose dependent manner (Figure 1A). For the rest of the work we used 1 nM BPA because is within the range of BPA levels in human serum and it is considered an environmentally relevant dose [7], [8].

**Figure 2:**
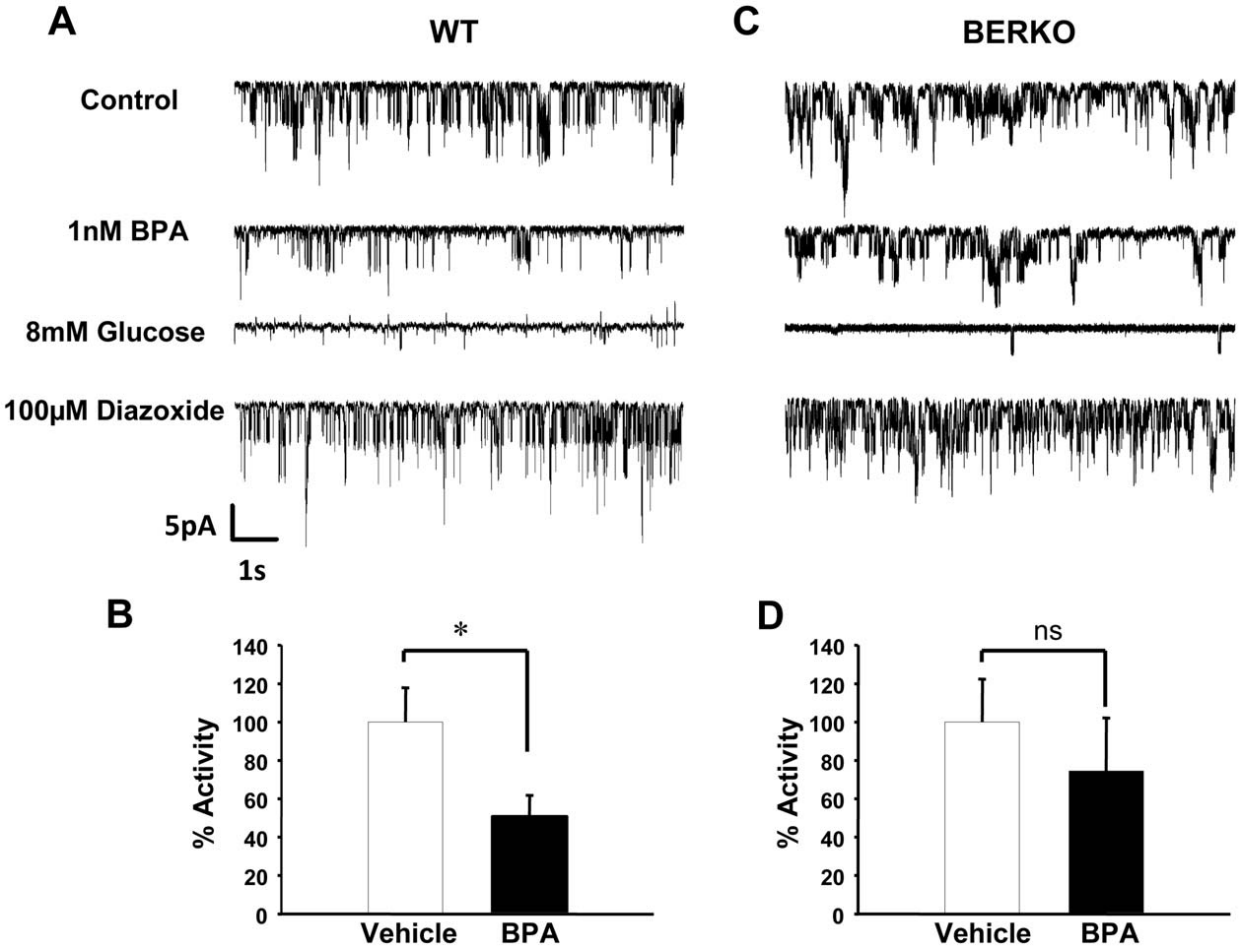
K_ATP_ channel activity in pancreatic β-cells from WT and ERβ−/− mice in presence of 1 nM BPA. A, BPA at 1 nM decreased K_ATP_ channel activity in pancreatic β-cells from WT mice. The records show channel activity before application of BPA (upper trace), 7 min after application of BPA (1 nM BPA trace), 8 min after application of 8 mM Glucose (8 mM glucose trace) and 2 min after application of diazoxide (100 µM diazoxide trace) (n = 7). B, percentage of activity of the K_ATP_ channels elicited by vehicle and 1 nM BPA. C, 1 nM BPA had no significant effect on K_ATP_ channel activity in pancreatic ERβ−/− mice. As in A, the records show K_ATP_ channel activity before application of BPA, 7 min after application of 1 nM BPA, 8 min after application of 8 mM glucose and 3 min after application of 100 µM diazoxide (n = 5 cells for 3 different mice). D, percentage of activity of K_ATP_ channels elicited by vehicle and 1 nM BPA. *, P<0.05 Student's test comparing 1 nM BPA with control.

Figure 2A shows, in a typical β-cell from wt mice, the basal activity of K_ATP_ channels (upper trace). Channel openings are represented by downward deflections, reflecting inward currents due to the high potassium content of the pipette. The criterion used for identification of K_ATP_ channels was their sensitivity to glucose, which highly decreases channel activity (Fig. 2A, 8 mM glucose trace) and diazoxide, a sulfonamide that opens K_ATP_ channels (Fig. 2A, 100 uM diazoxide trace). Both glucose and diazoxide sensitivity were tested in all records throughout this work. Figure 2A (BPA trace) demonstrates the decrease in the K_ATP_ channel activity elicited by BPA, 7 min after its application. On average, BPA decreases K_ATP_ channel activity by 49.3% (Fig. 2B).

To molecularly study the role of ERβ in BPA-induced rapid regulation of K_ATP_ channels, cell-attached patch recordings were performed in β-cells from ERβ−/− mice. In these cells K_ATP_ channel activity was not significantly altered after BPA application (Fig. 2C and D).

### BPA closes K_ATP_ channels in human β-cells

**Figure 3:**
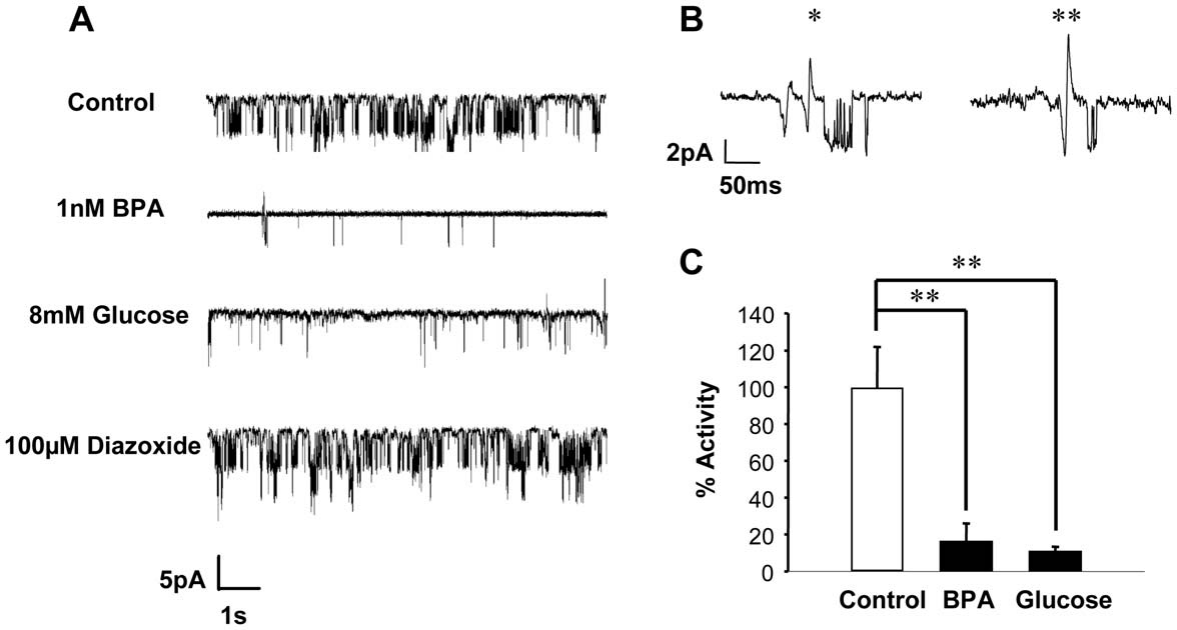
BPA regulation of K_ATP_ channel activity in human pancreatic β-cells. A, Application of 1 nM BPA decreased K_ATP_ channel activity in isolated human pancreatic β-cells in absence of glucose. The records show K_ATP_ channel activity in absence of glucose (vehicle trace), 5 min after application of 1 nM BPA, 8 min after application of 8 mM glucose and 3 min after application of 100 µM diazoxide. B, Both 8 mM glucose and 1 nM BPA depolarized membrane potential and action currents were generated (see * and ** in second and third traces in figure 2A). C, Percentage of activity of K_ATP_ channel elicited by vehicle, 1 nM BPA and 8 mM glucose in single β-cells (n = 5 cells). **, P<0.01 Student's test comparing 1 nM BPA and 8 mM glucose with control.

In order to analyze whether the rapid action of BPA on K_ATP_ channel activity occurred as well in human pancreatic β-cells, islets from human donors were disaggregated into single cells and single channel recordings were performed using the patch-clamp technique as performed in the previous subheading. Figure 3A shows a typical record in which 5 min after BPA application in the absence of glucose, there is a dramatic decrease in K_ATP_ channel activity by 83.4%, together with a depolarization of the membrane noticed by the decrease in the amplitude of the channel opening and the appearance of biphasic spikes (see * and ** in second and third traces and figure 3B). These biphasic spikes (action currents) were seen in all human cells exposed to either BPA or glucose and are action potentials seen via the capacitance and resistance of the patch and their recordings depend on the patch resistance [14], [30]. Remarkably, the response to BPA in the average of experiments is as high as that of 8 mM glucose (Fig. 3C). The action of BPA was mimicked by the ERβ agonist diaylpropionitrile (DPN) (Figure S1).

### BPA action on glucose induced [Ca^2+^]_i_ oscillations and insulin release is abolished in islets from ERβ−/− mice

**Figure 4:**
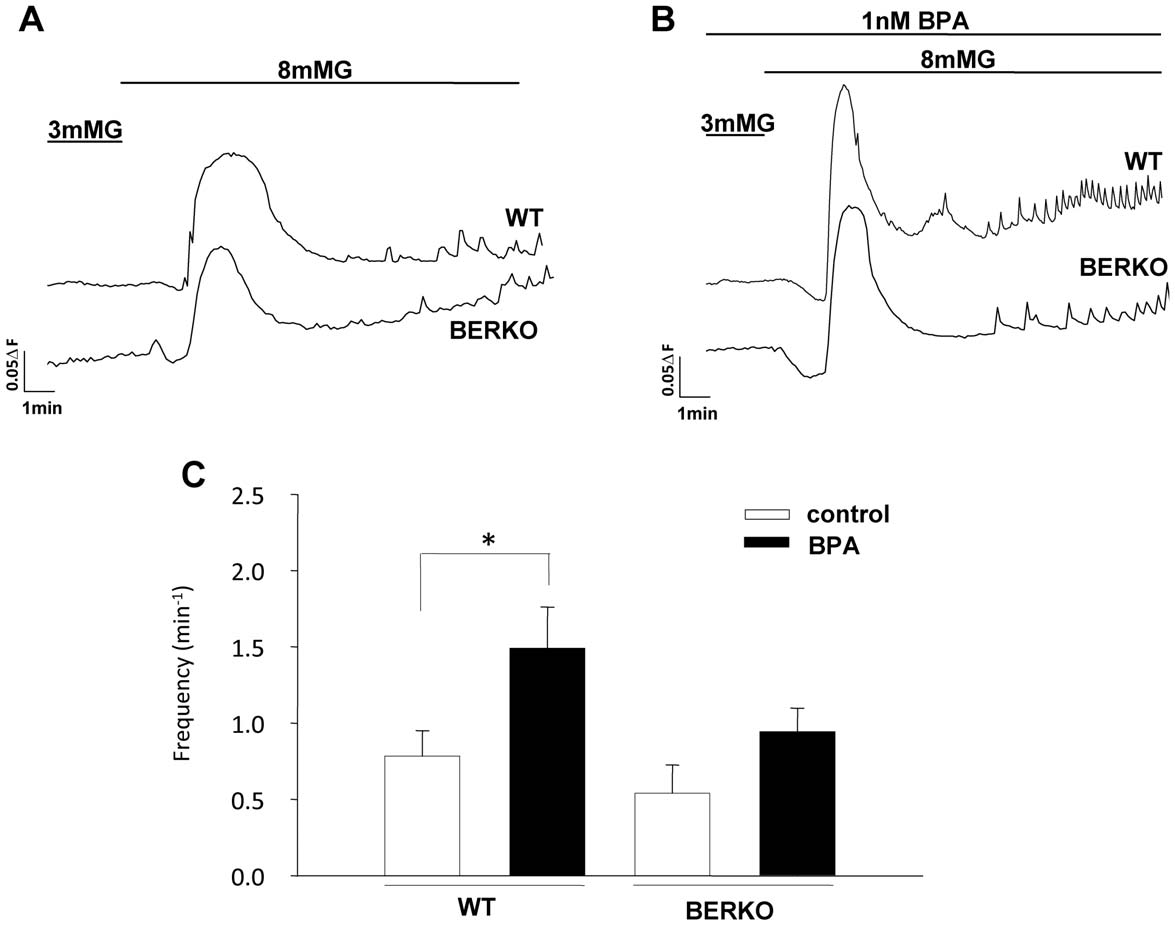
Effect of 1 nM BPA on glucose induced [Ca^2+^]_i_ oscillations in pancreatic β-cells from WT and ERβ−/− mice. A, Application of 8 mM glucose elicited a [Ca^2+^]_i_ transient followed by a plateau with [Ca^2+^]_i_ oscillations in pancreatic β-cells from both WT and ERβ−/− mice. B, application of 8 mM glucose in the presence of 1 nM BPA enhanced [Ca^2+^]_i_ oscillations frequency in WT islets but not in ERβ−/− islets. Recordings are representatives of at least 8 different islets from at least 4 different mice. C, frequency of [Ca^2+^]_i_ oscillations in pancreatic islets from WT and ERβ−/−mice in the absence of BPA (black bar) and in the presence of 1 nM BPA (white bar). Each point is the mean of at least eight different experiments. The analysis of the frequency was done during 5 min period, always taken when a steady state was reached, usually 8–10 min after 8 mM glucose application. *, P<0.05 Student's test.

**Figure 5:**
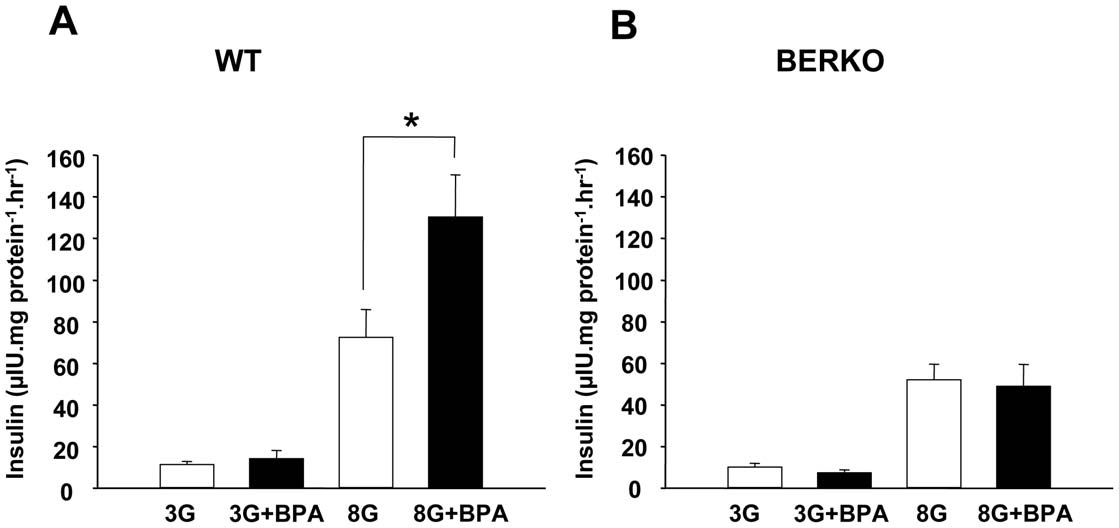
BPA effect on glucose-induced insulin secretion from isolated islets from WT and ERβ−/− mice. A, 1 nM BPA induced insulin secretion from WT islets exposed to 3 mM glucose and 8 mM glucose for 1 h. Note that 1 nM BPA action was significant only when 8 mM glucose was used. B, Same experiment as performed in A, but using ERβ−/− islets. Note that 1 nM BPA effect was abolished at 8 mM glucose (n = 5). *, P<0.05 Student's t-test comparing 8 mM glucose with 8 mM glucose in presence of 1 nM BPA.

It is well established that E2 rapidly induces Ca^2+^ signals and insulin secretion via ERβ in a process that depends on electrical activity [26]. To investigate whether activation of ERβ by BPA potentiated glucose-induced [Ca^2+^]_i_ signals and insulin secretion, we performed the experiments described in Figures 4 and 5. Islets loaded with the calcium fluorescence dye Fura-2 were imaged to monitor the [Ca^2+^]_i_ signal elicited by a change from a non stimulatory glucose concentration (3 mM) to an insulin stimulatory glucose concentration (8 mM). The response, as expected, was a [Ca^2+^]_i_ transient followed by a plateau with [Ca^2+^]_i_ oscillations. In the absence of BPA the frequency of [Ca^2+^]_i_ oscillations was similar in islets isolated from wt and ERβ−/− mice (Fig. 4A and C). However the frequency of [Ca^2+^]_i_ oscillations increased in islets exposed to 1 nM BPA compared to control in wt islets (Fig. 4B and C). It is of note that the BPA-induced increase in [Ca^2+^]_i_ oscillatory frequency is highly diminished in islets from ERβ−/− animals (Figure 4C). This experiment indicates that ERβ is involved in the BPA potentiation of glucose induced oscillatory activity.

In addition, BPA enhanced insulin release stimulated by 8 mM glucose islets from wt animals (Fig. 5A) but it had no effect in islets from ERβ−/− mice (Fig. 5B). Note that BPA had an effect only when a stimulatory glucose concentration (8 mM) was used; when BPA was applied together with 3 mM glucose no effect was observed.

### Insulinotropic action of BPA on human islets of Langerhans

**Figure 6:**
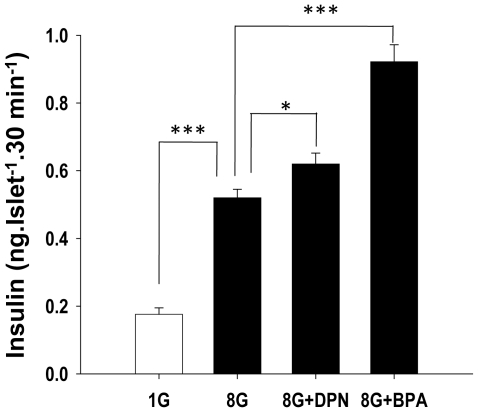
Insulinotropic action of BPA on isolated human islets of Langerhans. Stimulatory glucose concentration elicited insulin secretion that was enhanced almost 2 fold in presence of 1 nM BPA. Activation of ERβ by specific agonist DPN raised insulin secretion at 8 mM glucose concentration. *, P<0.05, **, P<0.01, ***, P<0.001 Student's t-test.

The rapid effect of 1 nM BPA on glucose stimulated insulin release was studied on human islets from two different donors. Islets responded to 8 mM glucose with an increase of insulin secretion. The action of 8 mM glucose was enhanced almost 2 fold by the presence of 1 nM BPA (Fig. 6). The ERβ agonist DPN applied at 1 nM concentration also enhanced insulin secretion, but to a lesser extent than 1 nM BPA (Fig. 6). This experiment indicates that environmentally relevant doses of BPA have an insulinotropic action on human islets of Langerhans.

## Discussion

The findings of this work using β-cells and whole islets of Langerhans from human donors demonstrate that the widespread endocrine disruptor BPA induces a rapid decrease of the activity of K_ATP_ channels, a key molecule in the stimulus secretion coupling of β-cells. Moreover, the present work demonstrates that environmentally relevant doses of BPA (1 nM) stimulated glucose-induced insulin secretion in human islets, giving a response which is almost twice the insulin release elicited by a stimulatory glucose concentration, 8 mM. These results together with previous observations that BPA diminished adiponectin release in human adipocytes [31], [32] support the premise that BPA has adverse effects on glucose metabolism in adult humans. Additionally, epidemiological evidence links BPA levels in urine with metabolic disorders in adult humans. These include type-2 diabetes, cardiovascular diseases and insulin resistance [33], [34].

In adult rodents, BPA exposure at environmentally relevant doses provokes insulin resistance and hyperinsulinemia in the fed state [35]. The hyperinsulinemia is not just a consequence of insulin resistance, because BPA directly regulates pancreatic insulin content and insulin release in isolated islet of Langerhans from the mouse [24]. Remarkably, this action occurred at low concentrations of BPA (1 nM) via activation of extranuclear ERα in a nonclassical manner that involves ERK1/2 activation and a nonmonotonic dose response [24]. The rapid effect described in the present work using β-cells from ERβ−/− mice demonstrates that ERβ is involved in the inhibition of K_ATP_ channel activity and in the regulation of glucose-induced [Ca^2+^]_i_ signals and insulin release. These actions imitate those of the natural hormone E2, and BPA and E2 are equally potent at 1 nM. It must be noted, however, that experiments performed in figures 2 and 4 using cells from ERβ−/− mice show a trend toward reduction in K_ATP_ channel activity and increased calcium signaling. In addition to ERα and ERβ, other non-classical membrane estrogen receptors (ncmER) involved in rapid BPA action have been described in β and α-cells [1], [23], [36]. GPR30 may be one of this ncmER because it exists in β-cells and is involved in cell survival [22], [37] and in glucose stimulated insulin secretion in response to supraphysiological concentrations of 17β-estradiol [22], [38]. Moreover, ncmERs may play a role in the supression of adiponectin release by BPA in mouse and human adipocytes [32].

**Figure 7:**
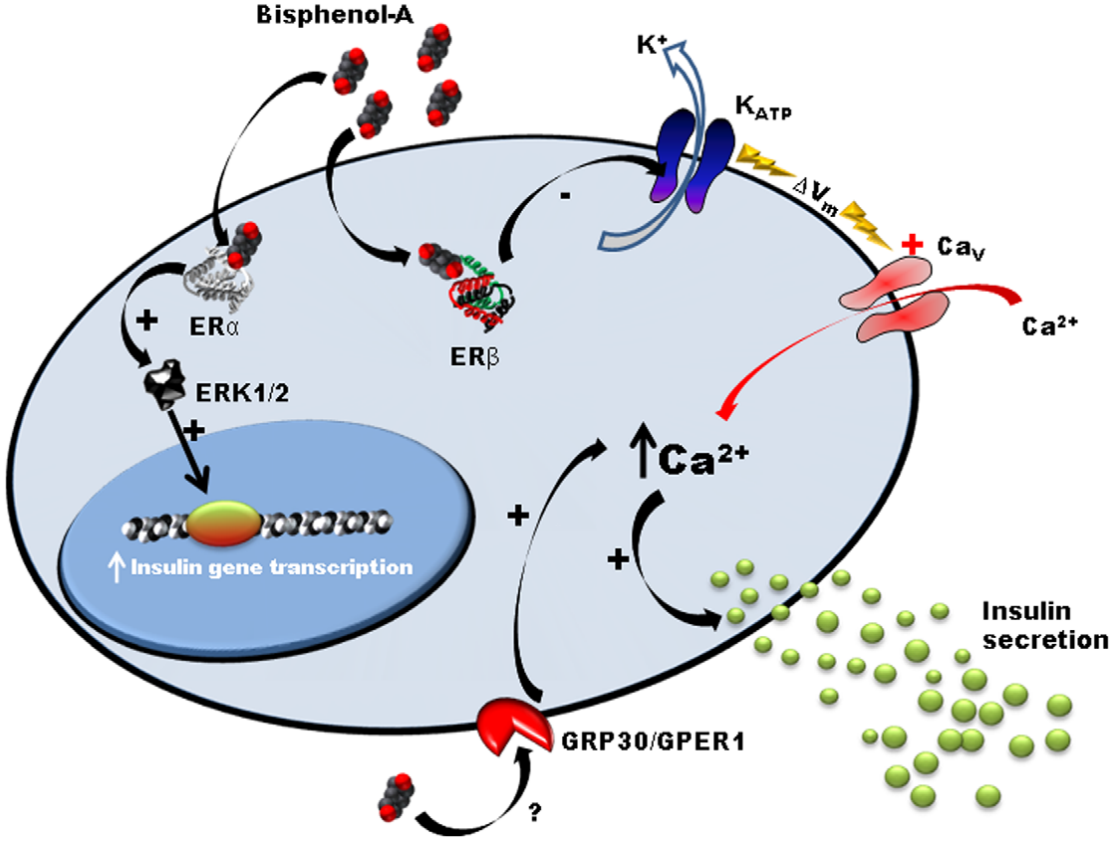
Model of BPA action on pancreatic β-cells. In the presence of stimulatory glucose concentrations, low concentrations of BPA rapidly decrease K_ATP_ channel activity through ERβ, enhancing glucose-induced [Ca^2+^]_i_ signals and insulin release. ERα is involved in the regulation of pancreatic insulin biosynthesis in response to BPA. In addition to ERβ, GPR30/GPER1 or another yet unidentified non-classical membrane estrogen receptor may participate in the insulinotropic effect of BPA on pancreatic β-cells. At the moment, this model applies to rodent beta cells. In humans, the receptors involved in the BPA regulation of K_ATP_ channel activity and insulin release are still undetermined.

BPA has been considered a weak estrogen because of its low binding affinity to both ERα and ERβ [39], as well as a low transcriptional activity through these ERE binding receptors [40]. ERα and ERβ control nuclear processes that include proliferation, apoptosis and migration exerting opposite effects in different tissues [41]. Nevertheless, it is fully accepted today that estrogen receptors do not work only in this classical manner but they signal as wellfrom extranuclear locations to exert a plethora of actions in different types of cells [37], [42]–[45]. In β-cells non-classical estrogen triggered pathways include regulation of insulin biosynthesis by ERα [24], [25], regulation of glucose stimulated insulin release by ERβ [26], improvement of cell survival by ERα and to a lesser extent by ERβ and GPR30 [37] and control of lipid synthesis by extranuclear ERα [45]. The nonclassical actions of estrogen receptors have main implications in energy balance as demonstrated using genetic rescue of nonclassical ER signalling in ER−/−mice [46]. The use of ERβ−/− mice in the present work and ERα−/− mice in a previous work [24] unequivocally demonstrates that both receptors acting though nonclassical pathways mediate BPA and E2 actions at equal concentrations (Fig. 7). Therefore, BPA cannot be considered a weak estrogen anymore, at least not in β-cells. Nevertheless, why the BPA effects occur at low doses when ERs are located in the extranuclear compartment is a matter of future research. It is possible that BPA binds differently to ERα and ERβ when they are located outside the nucleus. Also the recruitment of co-activators or co-repressors may be completely different [47]. Some of these effects may even be cell-specific. For instance, the insulinotropic action of BPA shown in this work occurs in the presence of a stimulatory glucose concentration of 8 mM. Under this condition most K_ATP_ channels are already closed and therefore the membrane resistance is high, so that a small current will elicit the depolarization of the membrane, electrical activity, Ca^2+^ signals and insulin release. This occurs in β-cells because K_ATP_ channels control the resting membrane potential and determine the electrical resistance of the cell [48]. This implies that the closure of a small number of K_ATP_ channels by BPA will produce an insulinotropic action.

In the case of human islets, BPA had a similar action to that in mice. It elicited a decrease in the K_ATP_ channel activity which was stronger than that in mice when the same dose of BPA was used. Moreover, its insulinotropic action was much stronger in human cells that in mice at the same dose. These results clearly demonstrate that environmentally relevant doses of BPA (1 nM) have an insulinotropic action on human islets. It is of note that the concentrations of BPA found in humans range from 0.7 to 20 nM [7], [8] and therefore the effect demonstrated here with islets from human donors may occur *in vivo* at usual levels of exposure. Rapid release of insulin *in vivo* in response to 10 µg/kg BPA was already reported in mice [35]. An excessive insulin signalling produced by an overstimulation of β-cells by BPA exposure may produce dyslipidemia resulting from effects in the liver and adipose tissue, and obesity and glucose intolerance [49]. In addition it may provoke insulin resistance in liver and skeletal muscle together with β-cell exhaustion, contributing to the development of type-2 diabetes [1]. Alterations of glucose and lipid metabolism by BPA in adults may constitute a significant hazard during pregnancy for both mothers and offspring as demonstrated in mice and rats [50], [51].

The ERβ agonist DPN also closed K_ATP_ channels and rapidly induced insulin release in human β-cells but to a smaller extent compared to mice. Given the scarcity of human islets we do not have enough data to definitely conclude that ERβ is the main receptor involved. The fact that ERβ mediates the insulinotropic action of BPA in mice does not rule out the possibility that other receptors such as GPR30/GPER1, which also bind BPA, may have different actions in mice and humans (fig. 7). Using GPR30 KO mice, it has recently been demonstrated that in addition to ERβ, GPR30 may also participate in the insulinotropic action of E2 in mice [38]. Moreover, it is known that activation of GPR30 by its agonist G1 is insulinotropic in mouse and human islets [22], [52] and regulates glucagon and somatostatin release from α and δ cells within the islet of Langerhans. Other not yet identified receptors acting on β- and other types of cells may also have a role in BPA signalling [31], [32], [53], [54].

The use of β-cells from genetically modified mice indicates that BPA cannot longer be considered a weak estrogen because it is equally potent to the natural hormone E2 when acting via nonclassical ER-mediated pathways. The rapid insulinotropic action of BPA described in mice also occurs in human islets and in a stronger manner to that in mice. This should have important implications for the policy of exposure of humans to BPA. There is an ongoing debate among environmental agencies worldwide about whether BPA can be considered a hazard for human health. One of the constant points of discussion is the relevance of extrapolation of animal results to humans. The demonstration that BPA at concentrations found in human serum alters human β-cell function strongly indicates that at least some of the adverse effects of BPA on glucose homeostasis described in mice may be translated to humans.

## Supporting Information

Figure S1 **Activation of ERβ by DPN decreased K_ATP_ channel activity in human pancreatic β-cells.** 1 nM DPN, specific agonist of ERβ, decreased K_ATP_ channel activity in isolated human pancreatic β-cells (1 nM DPN trace) compared with control conditions at 0 mM glucose concentration (vehicle trace). (n = 2).
(TIF)
