# Supplementary figures and images for: Rapid Insulinotropic Action of Low Doses of Bisphenol-A on Mouse and Human Islets of Langerhans: Role of Estrogen Receptor β
Source: PLoS One. 2012 Feb 8;7(2):e31109. doi: 10.1371/journal.pone.0031109 (PMC3275611; doi:10.1371/journal.pone.0031109)

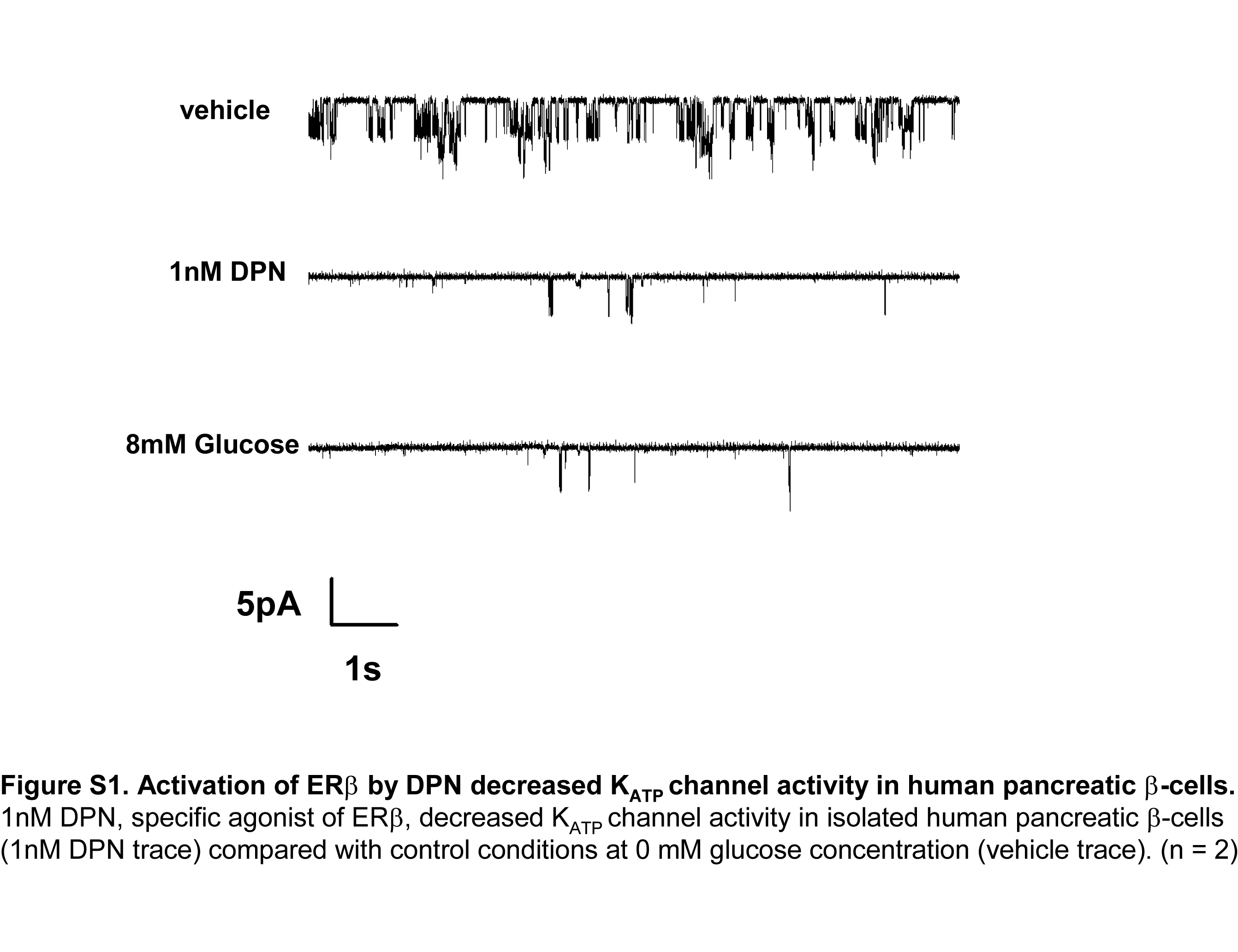

Supplement: Figure S1 — Activation of ERβ by DPN decreased KATP channel activity in human pancreatic β-cells. 1 nM DPN, specific agonist of ERβ, decreased KATP channel activity in isolated human pancreatic β-cells (1 nM DPN trace) compared with control conditions at 0 mM glucose concentration (vehicle trace). (n = 2). (TIF) [file pone.0031109.s001.tif]
